# BRCAsearch: written pre-test information and *BRCA1/2* germline mutation testing in unselected patients with newly diagnosed breast cancer

**DOI:** 10.1007/s10549-017-4584-y

**Published:** 2017-11-21

**Authors:** Martin P. Nilsson, Therese Törngren, Karin Henriksson, Ulf Kristoffersson, Anders Kvist, Barbro Silfverberg, Åke Borg, Niklas Loman

**Affiliations:** 1 Division of Oncology and Pathology, Department of Clinical Sciences, Lund University, Lund, Sweden; 2 Department of Hematology, Oncology and Radiation Physics, Skåne University Hospital, Lund, Sweden; 3 Department of Clinical Genetics, Laboratory Medicine Region Skåne, Lund, Sweden; 4 Department of Clinical Genetics, Lund University, Lund, Sweden

**Keywords:** Breast cancer, Unselected, Genetic testing, Counseling

## Abstract

**Purpose:**

To evaluate a simplified method of pre-test information and germline *BRCA1/2* mutation testing.

**Methods:**

In a prospective, single-arm study, comprehensive *BRCA1/2* testing was offered to unselected patients with newly diagnosed breast cancer at three hospitals in south Sweden (BRCAsearch, ClinicalTrials.gov Identifier: NCT02557776). Pre-test information was provided by a standardized invitation letter, but the patients could contact a genetic counselor for telephone genetic counseling if they felt a need for that. Noncarriers were informed about the test result through a letter. Mutation carriers were contacted and offered an appointment for in-person post-test genetic counseling.

**Results:**

During the period Feb 2, 2015–Aug 26, 2016, eight hundred and eighteen patients were invited to participate in the study. Through Jan 31, 2017, five hundred and forty-two (66.2%) of them consented to analysis of *BRCA1* and *BRCA2*. Eleven pathogenic mutations were found (*BRCA1*, *n* = 2; *BRCA2*, *n* = 9), corresponding to a mutation prevalence of 2.0%. Six out of 11 fulfilled the Swedish BRCA testing criteria, and 9 out of 11 fulfilled the NCCN testing criteria. None of the BRCA-associated tumors were of the luminal A-like subtype. Very few patients contacted us for telephone genetic counseling or practical questions, suggesting that a majority felt that the written pre-test information was sufficient for them to make a decision on testing.

**Conclusions:**

Streamlining the process of pre-test information, genetic testing, and delivery of test results was feasible and was associated with an uptake of genetic testing in 2/3 of the breast cancer patients.

## Introduction

The identification of a germline *BRCA1/2* mutation in a breast cancer patient is associated with potential benefits for herself as well as for her family members [1]. BRCA status could influence treatment decisions regarding local therapy and systemic therapy, and an oophorectomy performed after a diagnosis of breast cancer improves both breast cancer-specific survival and overall survival for mutation carriers [2–5]. Currently used selection criteria to merit genetic testing fail to detect up to half of the mutation carriers, and new methods for triaging patients to testing are needed [6, 7].

Over the last years, the rapid technical evolution and decreasing costs for genetic analyses have enabled testing on a much larger scale compared with what was previously feasible. Among other obstacles, the availability of genetic counselors is however a problem [7, 8]. Therefore, if genetic testing should be expanded to a larger number of individuals, then the counseling process needs to be simplified.

Recently, two large randomized trials on telephone genetic counseling for women at a high risk of BRCA mutations have been published [9, 10]. Telephone genetic counseling was cost effective and fulfilled the criteria for noninferiority to standard in-person counseling for all psychosocial and decision-making outcomes. The uptake of genetic testing was somewhat lower in the telephone arms: 28% versus 37% [9] and 84% versus 90% [10], respectively. A further simplification of the testing process is to offer written pre-test information instead of in-person or telephone counseling. No randomized trials on written pre-test information have been carried out in cohorts of unselected breast cancer patients, but a nonrandomized trial has suggested that it could be a feasible alternative to the standard procedure [11].

Taken together, currently available evidence suggests that the standard procedure of in-person genetic counseling could be modified in a cost-effective way without any perceivable negative impact on decisional conflict or psychosocial outcomes. The uptake rate is probably somewhat lower with written information or telephone counseling, but that drawback is outnumbered by the much larger number of patients that could be offered testing and by an increased number of identified mutation carriers.

We undertook a prospective, single-arm study of comprehensive *BRCA1/2* mutation screening in a consecutive series of unselected patients with newly diagnosed breast cancer (BRCAsearch, ClinicalTrials.gov Identifier: NCT02557776). Pre-test information was provided by a standardized invitation letter, but the patients could contact a genetic counselor for telephone genetic counseling if they felt a need for that. Here, we report the uptake of genetic testing, the prevalence of *BRCA1/2* mutations, the proportion of the mutation carriers that did not fulfill current criteria for BRCA testing, and how many of the patients that contacted us for questions, as well as some biological characteristics of the BRCA-associated breast tumors.

## Materials and methods

### Study cohort

Since late 2010, all patients with a newly diagnosed or strongly suspected invasive breast cancer in south Sweden are offered inclusion in an ongoing study called SCAN-B (Swedish Cancerome Analysis Network—Breast, ClinicalTrials.gov Identifier: NCT02306096) at the time of breast cancer diagnosis pre-surgery, or before start of preoperative medical treatment [12]. For consenting patients, a part of the tumor is sent to a research lab for RNA sequencing. SCAN-B is currently a biobank research study and the study results have no implications for the treatment decisions of individual participants. The main exclusion criteria for SCAN-B are the inability to understand written Swedish and severe psychological problems.

For reasons related to ethical permits and funding, only patients included in SCAN-B were eligible for inclusion in BRCAsearch. During the study period of BRCAsearch, 86% of all new invasive breast cancer cases at the participating hospitals were included in SCAN-B.

Inclusion criteria for BRCAsearch (all) are as follows: (i) the patient is included in the SCAN-B study; (ii) the patient is recently diagnosed with an invasive breast cancer; and (iii) the patient has signed an informed consent form for BRCAsearch.

Exclusion criteria for BRCAsearch (any) are as follows: (i) the patient is unable to understand the written information in Swedish; (ii) the patient is in a psychological state, due to chronic or temporary reasons, where one could suspect that information about the study substantially could be detrimental to the psychological well-being.

### Summary of study procedure for BRCAsearch

1. An invitation letter (Appendix 1) was given to the patient by the nurse at the regular visit to the surgeon a week after primary surgery or by the oncologist at the time of information about neoadjuvant chemotherapy. The invitation letter contained information about the study as well as possible implications of genetic testing, an informed consent form, psychosocial questionnaires, contact information to a genetic counselor, and a referral form for a blood sample for DNA extraction. The patient was invited to contact a genetic counselor for pre-test telephone counseling if she felt a need for more information.
2. If consent was given and the blood sample was sent in, *BRCA1* and *BRCA2* were analyzed (full sequencing; detailed in Appendix 2).
3. For patients who had not returned the consent form, one reminder was sent per mail approximately 2–4 months after the invitation letter.
4. Noncarriers were informed about the test result through a letter. Mutation carriers were telephoned and given a time for an appointment within one week at the Oncogenetic unit, Department of Clinical Genetics, Lund, Sweden, or, for patients who were at the time under adjuvant/neoadjuvant chemotherapy treatment, a visit was scheduled at the Department of Oncology.

According to the study protocol, patients that fulfilled the Swedish BRCA testing criteria should receive the invitation letter for BRCAsearch, but should also be referred for an assessment at the Department of Clinical Genetics, in order to enable testing of genes other than *BRCA1* and *BRCA2*.

**Fig. 1:**
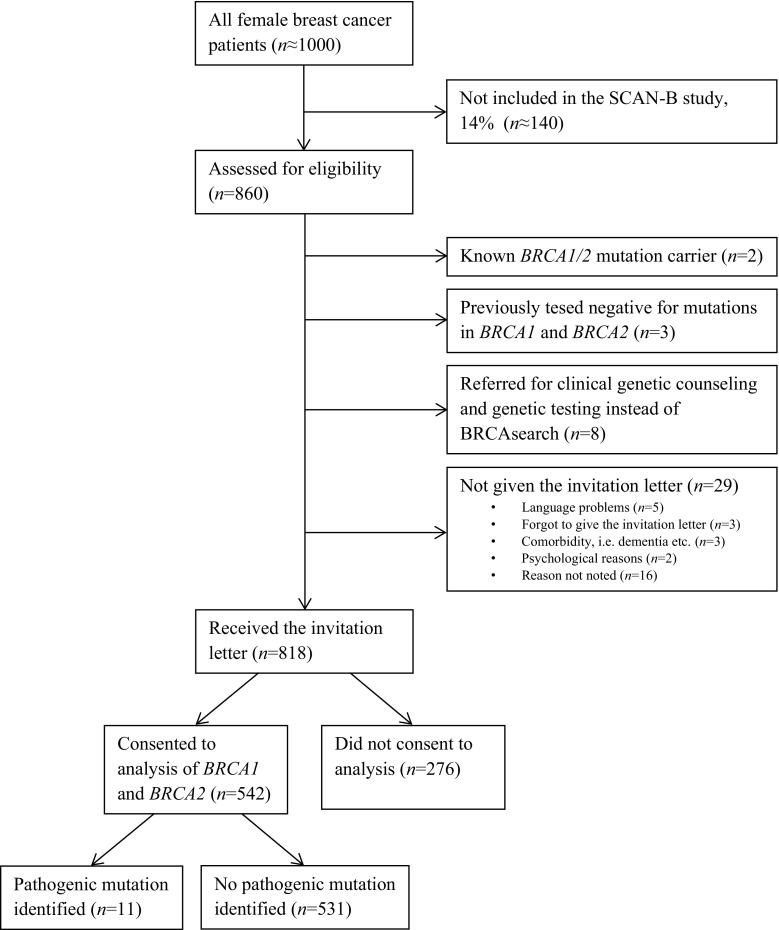
Flowchart of patient inclusion for BRCAsearch and genetic analyses

BRCAsearch enrolled patients from three hospitals in south Sweden: Helsingborg Hospital (February 2, 2015–August 26, 2016), Kristianstad Hospital (March 2, 2015–August 26, 2016), and Skåne University Hospital, Malmö (November 2, 2015–August 26, 2016). The targeted accrual was > 500 patients consenting to analysis, which was reached by August, 2016. The study flowchart is presented in Fig. 1. Information on BRCA sequence variant has been submitted to the Breast Cancer Information Core (https://research.nhgri.nih.gov/bic/).

## Results

### Uptake of genetic testing

The invitation letter was given to 818 patients with newly diagnosed breast cancer at three participating hospitals during the period Feb 2, 2015–Aug 26, 2016. The mean age at diagnosis was 63.6 years (median: 65.4 years; range: 26–94 years). Through Jan 31, 2017, five hundred and forty-two (66.2%) of them consented to analysis of *BRCA1* and *BRCA2* (Fig. 1). Consenting patients were younger at diagnosis than nonconsenting patients (mean: 61.8 vs. 67.1 years).

For nonconsenting patients, the study protocol dictated that a reminder should be sent by mail 2–4 months after the invitation to the study. However, due to practical reasons, the median time from the invitation to the study to the reminder was in fact 138 days (4.5 months). Out of 542 patients who consented to analysis, 459 (84.7%) consented without a reminder and 83 (15.3%) consented following a reminder.

### Time from breast cancer diagnosis to test result

The patients received the invitation letter at the first postoperative visit to the surgeon, usually a week after surgery. Patients treated with neoadjuvant systemic therapy instead received the invitation letter at the first visit to the medical oncologist (See “Materials and methods”). The median time from study invitation to study consent was 15 days. The median turnaround time for the mutation analyses at the laboratory was 48 days. The median time from the completion of the analysis until the letter was sent to the patient was 26 days. Consequently, the median time from breast cancer diagnosis to the delivery of the test result was approximately 3 months.

### Mutation analysis and prevalence of BRCA mutations

Eleven pathogenic mutations were found (*BRCA1*, *n* = 2; *BRCA2*, *n* = 9) in 542 tested patients, corresponding to a mutation prevalence of 2.0% (Agresti–Coull 95% confidence interval, CI: 1.1–3.6%). In addition, there were two patients who were assessed for eligibility that were already known *BRCA1/2* mutation carriers. Including those two patients in the analysis yielded a mutation prevalence of 2.4% (CI: 1.4–4.1%). Despite violating the study protocol, eight patients were referred for clinical genetic counseling and testing instead of receiving the invitation letter. As of yet, six of them have not been tested for BRCA mutations and two have tested negative.

### Characteristics of mutation carriers and BRCA-associated tumors

The mean age at diagnosis of the eleven mutation carriers previously not identified was 59.2 years (*BRCA1*: 52.1 years; *BRCA2*: 60.9 years). Four of them had previously been diagnosed with a breast cancer in the contralateral breast; consequently, the mean age at first breast cancer diagnosis was lower (53.1 years; median 49 years). Six out of 11 fulfilled the Swedish BRCA testing criteria [7], and 9 out of 11 fulfilled the NCCN BRCA testing criteria [13]. Tumor characteristics of BRCA-associated tumors are listed in Table 1. Regarding molecular subtypes (based on immunohistochemical surrogates according to modified St Gallen criteria [14]), no luminal A-like tumors were seen.

**Table 1:** Characteristics of mutation carriers (*n* = 11)

Mutation (HGVS)	Age at diagnosis (years)	St. Gallen subtype	TNM stage (AJCC 7th Edition)
BRCA2 c.9580_9581delCC	70	LumB HER2-	T1N0M0
BRCA2 c.8575_8575delC	49	LumB HER2-	T1N1miM0
BRCA1 c.1687C > T	46	Basal	cT2N0M0; ypTisN0^b^
BRCA2 c.5946_5946delT	68	LumB HER2-	T1N0M0
BRCA2 c.6267_6269delGCAinsC	63^a^	LumB HER2-	cT2N1M0; ypT0N0^b^
BRCA2 c.8953 + 1G > T	65	LumB HER2-	T1N0M0
BRCA2 c.4258_4258delG	47^a^	LumB HER2-	T1N2M0
BRCA2 c.4258_4258delG	72	LumB HER2-	T1N0M0
BRCA1 c.1687C > T	57^a^	LumB HER2-	T1N0M0
BRCA2 c.4258_4258delG	68^a^	LumB HER2-	cT2N1M0; ypT0N0^b^
BRCA2 c.5219_5219delT	40	LumB HER2+	T1N0M0

^a^Previous diagnosis of breast cancer in the contralateral breast; age refers to age at diagnosis of the second primary breast cancer

^b^Neoadjuvant chemotherapy

### Questions and genetic counseling

Only a small minority of the patients contacted us for questions related to genetic counseling (*n* = 14) or practical questions (*n* = 19). Out of 542 tested patients, eleven (2.0%) contacted us for questions related to genetic counseling and nineteen (3.5%) contacted us for practical questions, such as where to draw the blood sample and how long time the analysis would take.

## Discussion

We offered genetic testing of *BRCA1* and *BRCA2* to > 800 patients with newly diagnosed breast cancer and provided written pre-test information instead of in-person pre-test genetic counseling. Approximately 2/3 of the patients consented to testing. Very few contacted us for telephone genetic counseling or practical questions, suggesting that a majority of the patients felt that the written pre-test information was sufficient for them to make a decision on testing.

The prevalence of BRCA mutations among unselected breast cancer patients could be assessed in different ways within a single cohort, yielding slightly different results. Among the 542 patients tested within our study, the prevalence was 2.0%. Including the two patients that were already known mutation carriers at the time of breast cancer diagnosis, the prevalence was 2.4%. Factors increasing the likelihood of finding a mutation—such as younger age and family history of cancer—are probably more common among tested patients than among patients not consenting to analysis. Consequently, the prevalence of BRCA mutations among *all* breast cancer patients might be somewhat lower than these estimates. Another factor that needs to be taken into account is the uptake of prophylactic mastectomy among known mutation carriers. In the catchment area of the participating hospitals in our study, 102 known living female mutation carriers had undergone prophylactic mastectomy by Feb 2, 2015, when the study started (*BRCA1*, *n* = 67; *BRCA2*, *n* = 35), potentially preventing incident breast cancer in some mutation carriers during the period of time that the BRCAsearch study was open.

In countries with a high incidence of breast cancer, such as Sweden, the median age at diagnosis is higher than that in countries with a lower incidence of breast cancer. Since the median age of BRCA-associated breast cancer is lower than sporadic breast cancer, the overall prevalence of BRCA mutations among unselected breast cancer patients in high-incidence countries is probably lower than that in low-incidence countries. Very few studies, if any, have been carried out with comprehensive analysis of *BRCA1* and *BRCA2* in truly unselected breast cancer patients in high-incidence countries without strong founder mutations. Instead, data have been extrapolated from studies on selected cohorts and from studies that have used panel testing of previously identified mutations instead of full-length sequencing of both genes. The 2–3% prevalence of BRCA mutations found in our study is broadly in line with the prevalence estimates of these previous studies carried out in comparable populations [6, 11, 15, 16].

For ovarian cancer patients, the approval of PARP inhibitors for treatment of BRCA-deficient tumors has sparked much activity regarding methods of streamlining the process of genetic counseling, testing, and delivery of test results [17]. Also for breast cancer patients, there is currently an increasing interest in simplified protocols for genetic testing. The first randomized trial of written pre-test information in hereditary breast cancer was published in 2016 [18]. In this relatively small Australian study, 135 women with newly diagnosed breast cancer and a high (> 10%) likelihood of carrying a BRCA mutation were offered treatment-focused genetic testing (TFGT; testing prior to surgery that could inform surgical decisions). The patients were randomized to either standard care or a brief educational pamphlet instead of pre-test genetic counseling. Following testing, all patients received the test result at a face-to-face appointment. The intervention arm was cost effective and noninferior to the standard arm on the primary outcome decisional conflict [18].

No randomized trials on written pre-test information have been carried out in cohorts of unselected breast cancer patients. In a nonrandomized Norwegian study, that is very similar to our study in design, all newly diagnosed breast cancer patients were offered BRCA testing [11]. Written pre-test information was used instead of face-to-face counseling, and the uptake of testing was 45.4%, which is considerably lower than the uptake of testing in our study. The reasons for this difference in uptake are not clear, but could potentially be attributed to differences in the public knowledge of hereditary breast cancer, the wording in the written information, or other aspects of the study procedures. In the Norwegian study, the invitation letter was given to the patients at the time of diagnosis in order to have a result ready at time of primary surgery, a situation where the patient is faced with a lot of potentially overwhelming information regarding the diagnosis and treatment of the cancer. Also, in contrast to the Norwegian study, we sent a reminder to nonconsenting patients. Following the reminder, the uptake in our study increased from 56.1 to 66.2%.

If given a choice, it seems like a majority of patients eligible for testing would opt for a simplified mode of genetic counseling and delivery of test results. In a study from The Netherlands, 233 breast cancer patients referred for genetic counseling could choose between standard care and an intervention called “DNA-direct.” In DNA-direct, pre-test face-to-face genetic counseling was replaced by telephone and written and digital information. Of note, patients with psychological problems or difficulty with Dutch text were excluded. 161 patients (59%) opted for DNA-direct, of whom 90% were satisfied and would choose DNA-direct again (including 6 out of 8 BRCA mutation carriers) [19]. In another study from The Netherlands, counselees undergoing predictive testing of BRCA mutations or Lynch syndrome mutations all received standard pre-test genetic counseling, but were offered the choice to receive the test result either in-person or by a letter. A majority (69%) opted to receive the test result by a letter [20].

Over the last two decades, > 1500 families with pathogenic germline *BRCA1/2* mutations have been identified in Sweden. Most of these families have fulfilled clinical criteria for mutation testing. Of the mutations found, ~ 1030 are *BRCA1* and ~ 490 are *BRCA2* (ratio ~ 2:1). Founder mutations have been identified in Sweden but are not common; families carrying the five most recurrent mutations (all in *BRCA1* and found in 45 or more families) account for ~ 23% of the total number of families with a BRCA mutation (Å Borg, personal communication). In our present study, we notice that a majority of the mutations were *BRCA2* mutations. The different ratio could potentially be explained by the higher penetrance of ovarian cancer and younger age at breast cancer diagnosis in *BRCA1* carriers, making those families more likely to fulfill current BRCA testing criteria.

We also notice that none of the BRCA-associated tumors were of the luminal A-like subtype. Since treatment decisions regarding adjuvant chemotherapy are currently based mainly on molecular subtyping, 9 out of 11 mutation carriers received chemotherapy despite the fact that mutation status was not known at the time of primary treatment decisions. The median time from the date of breast cancer diagnosis to the delivery of the test result in our study was 3 months, meaning that most mutation carriers were informed about the test result while they were undergoing treatment with neoadjuvant or adjuvant chemotherapy. At the outset of the study, the intention was that the first information to the mutation carriers would be conveyed by a clinical geneticist. As the study progressed, it turned out that a more appropriate approach was to have a medical oncologist deliver the test results, since issues regarding additional systemic therapy (adjuvant PARP inhibitor trial), bilateral prophylactic mastectomy instead of postoperative radiotherapy for node-negative patients, and oophorectomy as a part of breast cancer treatment were often addressed already at this point of time.

There are limitations to our study. First, the nonrandomized study design precludes any solid comparisons with standard genetic testing procedures. Second, by only including patients that had previously been included in the SCAN-B biobank research study, 14% of all consecutive breast cancer patients were not eligible for inclusion and were not invited to participate in BRCAsearch. However, the exclusion criteria for SCAN-B and BRCAsearch were very similar, diminishing the importance of this selection bias, but also decreasing the proportion of the patients assessed for eligibility that were not given the invitation letter for BRCAsearch.

In summary, written pre-test information was a feasible way of streamlining the process of genetic testing in newly diagnosed breast cancer patients. Future studies evaluating potential psychosocial impacts of proactive and simplified procedures of genetic testing are needed, preferably with randomized study designs. Also, the penetrance of *BRCA1/2* mutations identified within cohorts of unselected patients needs further study.

## Appendix 1: the invitation letter

### An invitation to participate in a study about hereditary breast cancer

#### Introduction

You are already participating in a study (SCAN-B) that is dealing with breast cancer and causes of breast cancer. We would like to ask you if would like to participate in an optional complementary study, aiming at finding out the frequency of hereditary breast cancer. The complementary study is called BRCAsearch.

#### Background

There are two genes, breast cancer gene 1 (BRCA1) and breast cancer gene 2 (BRCA2), that in rare cases could have an inborn error, a mutation. A mutation in any of these genes might explain why a certain person is affected with breast cancer. In most cases, women with a mutation have inherited the mutation from their mother or their father. In these cases, there is frequently—but not always—breast cancer in the family. Among women who get breast cancer in Sweden, we estimate that 2–4%, meaning 2–4 out of 100 women with breast cancer, are carriers of a mutation in BRCA1 or BRCA2.

If you would like to participate in BRCAsearch, we will find out whether or not you are a carrier of a mutation in BRCA1 or BRCA2. The analysis is carried out on a blood test. You will be informed about the results of the analysis.

#### What are the consequences if one is a carrier of a mutation in BRCA1 or BRCA2?

Women with a mutation in BRCA1 or BRCA2 are at a high risk for breast cancer. Among women without a mutation, the risk of breast cancer throughout life is about 1 out of 10, that is 10%. Among women with a mutation in BRCA1 or BRCA2, the risk of breast cancer is instead 5–8 out of 10, that is 50–80%.

Women with a mutation in BRCA1 or BRCA2 who have already had breast cancer do not have an increased risk of recurrence of the disease compared to women without a mutation. However, women with a mutation have an increased risk of breast cancer in the other breast. Therefore, these women are followed carefully with various breast examinations. In addition, the risk of ovarian cancer is increased in women with a mutation. Up to every second woman with a mutation is affected by ovarian cancer throughout life. That risk is much higher than in the general population, where the risk of ovarian cancer is approximately 1%.

#### What does this mean to you?

If you would like to participate in BRCAsearch you should sign the consent form (printed on pink paper) and return it in the enclosed reply envelope. In addition to that, you or your nurse should write your name and your personal identification number on the enclosed blood test referral form (yellow). After having done that, you take a blood test at your hospital or local health center. We will then analyze your blood test and search for mutations in BRCA1 and BRCA2. The analysis takes up to 3–4 months to complete. After the analysis is completed, we will notify you with a letter if you are not a carrier of a mutation. If we find that you are a carrier of a mutation, we will instead call you on the telephone number that you have specified below. After we have called, you will be offered a visit at the hospital in Lund for further information. We will also call you if we find a change of uncertain significance in any of these two genes. Such a change might require further investigations to clarify its significance.

It does not cost anything to participate in the study, but neither is any economic compensation given for the participation. If you do not want to participate in the study you do not have to do anything at all, and in that case you should not sign the consent form.

#### Are there any disadvantages or risks associated with the participation in BRCAsearch?

There are no medical risks. On the other hand, the awareness of one being a mutation carrier could cause feelings of anxiety and depression. For instance, one could feel sad about the risk that one’s children might have inherited the mutation.

#### Are there any advantages associated with the participation in BRCAsearch?

If it turns out that you belong to the small minority of breast cancer patients that have a mutation in BRCA1 or BRCA2, it is an advantage to know about that, since one could then take prophylactic measures in order to decrease one’s risk of cancer, for instance extra surveillance regarding the breasts and prophylactic surgery regarding the ovaries.

#### Biobank and personal data

Your blood test will be stored in Skåne County Counsil’s Biobank. Without specifying any reason, you can at any time request that the blood test or other information about you must not be used in the future. In the study, your personal data will be managed with computers. The Skåne County Council is the controller of personal data in accordance with the Law of Personal Data. When you consent to participation in the study, you also consent to processing of personal data. Only personnel involved in the study have access to the data. The personnel are bound by professional secrecy. You have the right to obtain information about the processing of personal data according to the Law of Personal Data. You do that by writing to the Personal Data Protection Officer, Skåne County Council, SE-291 89 Kristianstad. The request must be autographically signed.

#### Voluntariness

The participation in BRCAsearch is optional. Without specifying any reason, you have the right to discontinue your participation at any time, by contacting us by phone on the number below or by mail to the address below.

#### The following persons are responsible for the study

Dr Niklas Loman, Senior Consultant, Department of Oncology, Skåne University Hospital and Lund University.

Dr Ulf Kristoffersson, Senior Consultant, Department of Clinical Genetics, Lund.

Dr Martin Nilsson, Department of Oncology, Skåne University Hospital and Lund University.

Barbro Silfverberg, Genetic Counselor, Department of Clinical Genetics, Lund.

#### Our contact information

If you have questions about BRCAsearch, or if you wish to have further information before you decide on whether you would like to participate or not, you are welcome to contact us per telephone, mail, or e-mail.

## Appendix 2: sequencing and variant calling

### Library preparation, hybridization capture, and MPS sequencing

DNA samples were quantified using the Qubit system (Life Technologies, Carlsbad, CA, USA). Two µg of DNA was fragmented using the Covaris S2 Ultrasonicator (Covaris, Woburn, MA, USA). SureSelectXT Custom 3-5.9 Mb library kit (Agilent Technology, Santa Clara, CA, USA) was used to capture DNA fragments from BRCA1, BRCA2, and 62 other target genes. Eight samples were pooled before capture and the molarity of the library pool after capture was determined using concentration measured by Qubit and DNA fragment size distribution measured on a Bioanalyzer (Agilent). Sequencing was performed on the Illumina HiSeq 2500 (Illumina, San Diego, CA, USA) with 2 × 94 to 2 × 101 bp paired end reads.

### Analysis of sequencing data

Format conversion and demultiplexing of raw Illumina sequencing data were done using Picard ExtractIlluminaBarcodes and IlluminaBasecallsToSam (https://broadinstitute.github.io/picard/). Sequence reads were aligned to the human reference genome hs37d5ss (1000 genomes with decoy sequences) using NovoAlign (http://www.novocraft.com). PCR duplicates were identified and marked for exclusion in subsequent analyses using Picard MarkDuplicates. Base quality scores were recalibrated and indels realigned using the Genome Analysis Tool Kit (GATK, McKenna et al., 2010). Genetic variants were identified and genotypes called with GATK UnifiedGenotyper with a call confidence cutoff of 10. Variants were annotated for their effect on protein coding transcripts using SnpEff and Annovar using RefSeq reference transcripts. Variants in target genes other than the ones under study were removed from the variant list files. Variants affecting coding exons and 20 bp of adjacent introns of BRCA1 and BRCA2 were evaluated for pathogenicity.

### Copy number variation (CNV) analysis

Copy number variants affecting one or more exons of the target genes were identified using an in-house method based on the number of read pairs in short windows over the target regions (window size 240–480 bp). Read coverage was normalized based on GC, capture probe density, and mean read depth for each sample. The ratio of normalized coverage for the sample and an average of multiple normal samples (baseline) were computed. Potential deletions were detected as one or more consecutive windows with a normalized coverage ratio below 0.75 and duplication as consecutive windows with normalized coverage above 1.25. Potential deletions and duplications were inspected in IGV to identify breakpoints.

### Sanger sequencing

Any bases of coding exons and 20 bp of adjacent introns of BRCA1 and BRCA2 that were not covered by at least 30 reads were Sanger sequenced. All variants affecting coding exons and 20 bp of adjacent introns of these genes were also verified using Sanger sequencing. Samples were prepared using BigDye Terminator v1.1 Cycle Sequencing Kit (Applied Biosystems, Foster City, CA, USA) and sequenced on a 3130xl Genetic Analyzer (Applied Biosystems). Chromatograms were evaluated using Sequencher 5.0 (Gene Codes Corporation, Ann Arbor, MI USA).

### Assessment of variant pathogenicity

BRCA gene sequence variants were classified into the IARC 5-tiered system as described by the ENIGMA (Evidence-based Network for the Interpretation of Germline Mutant Alleles) consortium (http://enigmaconsortium.org/). Within the current study, only class 5 (pathogenic) and class 4 (likely pathogenic) variants were reported back to patients. In short, this would include coding sequence variants that lead to a premature termination codon (i.e., nonsense or frameshift alteration) which disrupts the expression of an important functional protein domain. It also includes variants likely to alter splicing (i.e., positions IVS ± 1 and IVS ± 2) or confirmed to do so by in vitro allele-specific assays. It includes copy number deletions or duplications of one or more exons that give rise to frameshifts and/or disruption of a functionally important domain. Finally, it includes missense variants with a high, i.e., > 0.95, probability of pathogenicity in multifactorial likelihood models, including results from functional assays. All 11 variants classified as pathogenic in this study have been reported elsewhere (https://www.ncbi.nlm.nih.gov/clinvar/).
